# Hypersensitivity Reaction to Ferric Carboxymaltose in a Postpartum Patient After Emergency Cesarean Section: A Case Report

**DOI:** 10.7759/cureus.117787

**Published:** 2026-10-11

**Authors:** Natasha Gupta, Vidhi Sambyal, Meeta Gupta, Shivani Sharma, Vishakha Gupta, Vanessa Ravel

**Affiliations:** 1 Obstetrics and Gynecology, All India Institute of Medical Sciences, Vijaypur, Jammu, IND; 2 Public Health, Independent Research, Chennai, IND

**Keywords:** ferric carboxymaltose, gestation, hypersensitivity, intravenous iron, postpartum anemia

## Abstract

Ferric carboxymaltose (FCM) is a common treatment for the immediate management of postpartum iron deficiency anemia and is generally well tolerated. However, rare instances of hypersensitivity reactions, including delayed reactions, may occasionally occur. This case report presents a 32-year-old post-cesarean section patient who developed bilateral lower-limb edema, generalized pruritus, and a GI complaint (loose stools) two hours after administration of FCM, without hemodynamic instability or respiratory distress. The reaction was considered probable based on the temporal association between FCM administration and symptom onset, improvement after treatment with corticosteroids and antihistamines, and the absence of an alternative explanation. Causality assessment using the WHO-UMC Causality Assessment System and the Naranjo Adverse Drug Reaction Probability Scale further supported a probable drug-related reaction. The symptoms subsequently resolved with treatment. This case highlights that FCM may rarely present with delayed and atypical hypersensitivity manifestations in postpartum patients, emphasizing the need for post-infusion observation and early recognition.

## Introduction

Postpartum anemia is a significant contributor to maternal morbidity, particularly in developing countries, where nutritional iron deficiency remains highly prevalent. The WHO defines postpartum anemia as a hemoglobin concentration of <110 g/L at one week postpartum and <120 g/L at eight weeks postpartum [1]. Oral iron tablets are frequently recommended for treatment but may be associated with GI side effects, poor adherence, and inadequate absorption, particularly in moderate-to-severe anemia [2].

The administration of IV iron provides a rapid and effective alternative. Ferric carboxymaltose (FCM), a third-generation non-dextran IV iron product, can be administered in large amounts over a short period and is generally well tolerated, with an overall favorable safety profile [3]. Nevertheless, despite its overall safety, very rare cases of hypersensitivity and anaphylactoid reactions have been reported, which should not be overlooked [3,4]. Although most reported severe reactions involve respiratory or cardiovascular instability, delayed and atypical presentations may remain unnoticed due to the similarity of signs to postoperative conditions in postpartum women. In this case report, we describe a delayed-onset, mild hypersensitivity reaction characterized by bilateral lower-limb edema, generalized pruritus, and GI symptoms, including loose stools, following the administration of FCM.

## Case presentation

A 32-year-old woman, gravida 4, para 2, living 1, abortion 1 (G4P2L1A1), was admitted to the labor ward in the Department of Obstetrics and Gynecology at 36 weeks of gestation with preterm labor. She had a history of one previous preterm cesarean section. Her pregnancy was complicated by gestational diabetes mellitus and hypothyroidism, managed with medical nutrition therapy and levothyroxine 25 µg once daily. She also had mild anemia (preoperative Hb 9.4 g/dL).

She underwent an emergency preterm lower segment cesarean section (LSCS) under spinal anesthesia because of her history of a previous preterm LSCS. The intraoperative and immediate postoperative periods were uneventful, and no blood transfusion was required. There was no history of uterine atony or postpartum hemorrhage. A live preterm neonate weighing 2.34 kg was delivered, with Apgar scores of 7/10, 8/10, and 9/10 at 0, 5, and 10 minutes, respectively. On postoperative evaluation, the patient was hemodynamically stable. Clinical examination revealed mild pallor without icterus, cyanosis, or edema. There was no tachycardia. There was no history of drug allergy, atopy, or asthma. Systemic examination was otherwise unremarkable.

Laboratory investigations revealed a hemoglobin level of 9.4 g/dL and a serum ferritin level of 8.39 ng/mL. Peripheral smear examination showed microcytic hypochromic red blood cells. Liver function tests, renal function tests, and coagulation profile were within normal limits. In view of her low iron stores, IV FCM was planned (Table 1).

**Table 1:** Preoperative hematological and biochemical investigations of the patient along with their normal reference ranges. Reference ranges are the normal ranges established by the institution’s laboratory and may vary according to the laboratory and analytical method used. ALT, alanine aminotransferase; AST, aspartate aminotransferase; Hb, hemoglobin; LDH, lactate dehydrogenase; MCH, mean corpuscular hemoglobin; MCHC, mean corpuscular hemoglobin concentration; MCV, mean corpuscular volume; SGOT, serum glutamic-oxaloacetic transaminase; SGPT, serum glutamic-pyruvic transaminase; TLC, total leukocyte count

Parameters	Patient value	Reference ranges
Hemoglobin (g/dL)	9.4	12-15
TLC (10³/mm³)	9.73	4-10
Serum ferritin (ng/mL)	8.39	15-150
RBC count (10⁶/mm³)	3	3.8-4.8
Serum iron (µg/dL)	26	50-170
MCV (fL)	72	82-100
MCH (pg)	25	27-32
MCHC (g/dL)	27	33-35
Urea (mg/dL)	16	13-40
Serum creatinine (mg/dL)	0.6	0.6-1.1
Serum albumin (g/dL)	4	3.5-5.2
Total bilirubin (mg/dL)	0.6	0.3-1.0
SGOT (AST) (U/mL)	16	<34
SGPT (ALT) (U/mL)	11	<31
LDH (U/L)	352	225-450

Adverse drug reaction

On postoperative day 3, the patient received 1 g of FCM intravenously, diluted in 200 mL of normal saline and administered over 15-20 minutes. Approximately two hours after completion of the infusion, the patient developed swelling over both feet and legs, generalized itching, and one episode of loose stools. It was not associated with exanthem or urticaria.

Postoperative edema and fluid shifts were considered as close differential diagnoses; however, the patient had no edema on the pre-infusion examination, and the bilateral lower-limb swelling developed abruptly after FCM administration, together with generalized pruritus and a loose stool, and resolved shortly after treatment. Hypertensive disorders of the postpartum period were also considered; however, there was no documented hypertension, and renal and liver function tests were within normal limits. Deep vein thrombosis was considered less likely because the edema was bilateral, without reported respiratory symptoms, and there was no clinical evidence suggesting an acute thromboembolic event. Taken together, the close temporal relationship, associated systemic symptoms, absence of preexisting edema, and improvement following treatment supported FCM-associated hypersensitivity. There was no associated respiratory distress, hypotension, or loss of consciousness. Based on the clinical presentation and the temporal relationship with FCM administration, a diagnosis of a hypersensitivity reaction to FCM was considered.

Management

The patient was immediately treated with Inj. hydrocortisone 100 mg IV stat and Inj. pheniramine maleate (Avil) 75 mg IV stat. The patient responded well to treatment, with gradual resolution of her symptoms. No recurrence or progression of symptoms was observed. Table 2 outlines the timeline of clinical events.

**Table 2:** Timeline of clinical events. FCM, ferric carboxymaltose

Time points	Clinical events
Day 0	Emergency lower segment cesarean section performed
Postoperative days 1-2	Stable postoperative recovery
Postoperative day 3	IV FCM (1 g) administered
Two hours post-infusion	Onset of bilateral leg swelling, pruritus, and loose stools
Immediately after symptom recognition	Administration of hydrocortisone and pheniramine
Within hours	Resolution of symptoms
48 hours after the reaction	Patient remained stable and was discharged on oral iron therapy.

Subsequently, the patient was managed with oral iron supplementation. At discharge, she was hemodynamically stable, her surgical wound was healthy, and uterine involution was appropriate. Both mother and baby were discharged in satisfactory condition with advice for follow-up in the outpatient department.

## Discussion

FCM is increasingly favored for the management of postpartum iron deficiency anemia because of its ability to rapidly replenish iron stores and its convenience, which may improve adherence compared with oral iron therapy. IV iron is particularly useful in the postpartum period when oral iron is poorly tolerated or ineffective [1-2,5].

Hypersensitivity reactions to IV iron are uncommon and are generally non-IgE-mediated, often attributed to complement activation-related pseudoallergy (CARPA) rather than true anaphylaxis. One proposed mechanism is the activation of the complement system by iron-carbohydrate nanoparticles, leading to the generation of anaphylatoxins such as C3a and C5a. These mediators can stimulate mast cells and basophils and result in the release of vasoactive mediators, producing clinical manifestations resembling anaphylaxis. However, the precise pathophysiology of hypersensitivity reactions to IV iron remains incompletely understood, and an IgE-mediated mechanism cannot be entirely excluded in individual cases [6,7]. Although FCM is associated with a lower incidence of serious reactions compared with older iron formulations, adverse effects such as pruritus, edema, GI symptoms, and, rarely, severe anaphylaxis have been reported [4]. Delayed hypersensitivity reactions after FCM appear to be uncommon and are described mainly in individual case reports or small clinical series rather than in large studies with a clearly defined incidence.

The European Medicines Agency recommends that all IV iron infusions be administered in settings with immediate access to resuscitation facilities and that patients be observed during and after infusion because of the potential for hypersensitivity reactions [8]. In the present case, the onset of symptoms approximately two hours post-infusion emphasizes the importance of post-infusion monitoring. The patient’s symptoms resolved following treatment with antihistamines and corticosteroids, with no subsequent progression to anaphylaxis. Although IV iron is associated with fewer GI side effects than oral iron, it carries a risk of hypersensitivity reactions, including rare but potentially life-threatening anaphylaxis. Therefore, administration should be undertaken by appropriately trained healthcare workers who can monitor patients for signs of adverse reactions and respond promptly with appropriate emergency management. Although severe anaphylactic reactions are rare, robust monitoring and pharmacovigilance systems should be established to document adverse events and monitor the safety of IV iron as its use is scaled up.

The suspected adverse drug reaction was identified and reported to the Pharmacovigilance Programme of India through the institutional Adverse Drug Reaction Monitoring Centre, thereby contributing to the national pharmacovigilance effort. This reporting of adverse drug events helps in pharmacovigilance efforts and highlights the necessity to be cautious regarding IV iron products in postpartum patients.

Causality assessment using the WHO-UMC Causality Assessment System and the Naranjo Adverse Drug Reaction Probability Scale (score = 7) classified the reaction as “probable” based on a clear time relationship, resolution of the reaction after cessation of the drug, and lack of other explanations [9,10]. The score was supported by the appearance of symptoms following FCM administration and improvement of the patient’s symptoms following treatment and discontinuation of the suspected drug, but it lacked any attempt to re-administer the drug. Preventive measures should include acquiring a thorough history of any past drug allergy, atopy, asthma, or prior reactions to iron preparations. This product should be administered only under monitored conditions where resuscitation facilities are readily available, together with drugs such as adrenaline, antihistamines, corticosteroids, and oxygen therapy. Vital signs must be taken before, during, and after the injection, with special attention during the first 15-30 minutes and subsequent monitoring for delayed reactions. Routine premedication is not universally recommended, as it may mask early symptoms, and its role in preventing hypersensitivity reactions has not been established.

## Conclusions

FCM is generally considered safe; however, clinicians should remain vigilant for hypersensitivity reactions despite adherence to recommended administration and monitoring practices. Delayed-onset reactions to FCM may occur in the absence of anaphylaxis. In patients following cesarean delivery, the occurrence of bilateral lower-limb swelling associated with pruritus and GI disturbances should not be ignored. Although most hypersensitivity reactions occur during or shortly after infusion, this case highlights the need for continued observation beyond the immediate post-infusion period, particularly when symptoms may have a delayed onset. Close clinical observation following FCM administration, along with prompt recognition and symptomatic management of suspected reactions, may facilitate timely intervention.
